# Secretory Leukocyte Protease Inhibitor (SLPI) Is, like Its Homologue Trappin-2 (Pre-Elafin), a Transglutaminase Substrate

**DOI:** 10.1371/journal.pone.0020976

**Published:** 2011-06-07

**Authors:** Kévin Baranger, Marie-Louise Zani, Valérie Labas, Sandrine Dallet-Choisy, Thierry Moreau

**Affiliations:** 1 Inserm U618 “Protéases et Vectorisation Pulmonaires”, IFR 135 Imagerie Fonctionnelle, University of Tours, Tours, France; 2 Laboratoire de spectrométrie de masse, Plateau d'analyse intégrative des biomarqueurs cellulaires et moléculaires, INRA, Tours-Nouzilly, France; Consejo Superior de Investigaciones Cientificas, Spain

## Abstract

Human lungs contain secretory leukocyte protease inhibitor (SLPI), elafin and its biologically active precursor trappin-2 (pre-elafin). These important low-molecular weight inhibitors are involved in controlling the potentially deleterious proteolytic activities of neutrophil serine proteases including elastase, proteinase 3 and cathepsin G. We have shown previously that trappin-2, and to a lesser extent, elafin can be linked covalently to various extracellular matrix proteins by tissue transglutaminases and remain potent protease inhibitors. SLPI is composed of two distinct domains, each of which is about 40% identical to elafin, but it lacks consensus transglutaminase sequence(s), unlike trappin-2 and elafin. We investigated the actions of type 2 tissue transglutaminase and plasma transglutaminase activated factor XIII on SLPI. It was readily covalently bound to fibronectin or elastin by both transglutaminases but did not compete with trappin-2 cross-linking. Cross-linked SLPI still inhibited its target proteases, elastase and cathepsin G. We have also identified the transglutamination sites within SLPI, elafin and trappin-2 by mass spectrometry analysis of tryptic digests of inhibitors cross-linked to mono-dansyl cadaverin or to a fibronectin-derived glutamine-rich peptide. Most of the reactive lysine and glutamine residues in SLPI are located in its first N-terminal elafin-like domain, while in trappin-2, they are located in both the N-terminal cementoin domain and the elafin moiety. We have also demonstrated that the transglutamination substrate status of the cementoin domain of trappin-2 can be transferred from one protein to another, suggesting that it may provide transglutaminase-dependent attachment properties for engineered proteins. We have thus added to the corpus of knowledge on the biology of these potential therapeutic inhibitors of airway proteases.

## Introduction

A characteristic of inflammation is the release of serine proteases (NSPs) from the azurophil granules of activated neutrophils. These enzymes, including leukocyte elastase (HNE), proteinase 3 (Pr3) and cathepsin G (CatG), are involved in the proteolytic degradation of extracellular matrix proteins like elastin. They are therefore believed to play a key role in inflammatory tissue-destroying diseases of the lungs, such as chronic obstructive pulmonary disease (COPD), acute lung injury and cystic fibrosis (see [1] for a review). The activities of NSPs in the human lung are mainly regulated by endogeneous protease inhibitors like the serpin α1-PI and canonical inhibitors belonging to the chelonianin family of protease inhibitors. This latter family, assigned to Family I17 Clan IP in the MEROPS database (http://merops.sanger.ac.uk), includes secretory leukocyte protease inhibitor (SLPI), elafin and its precursor trappin-2 (or pre-elafin) [2] from which elafin is proteolytically released, possibly by mast cell tryptase [3]. SLPI is a potent inhibitor of leukocyte elastase and cathepsin G, whereas elafin and trappin-2 preferentially target leukocyte elastase and proteinase 3 (see [2] for a review). Trappin-2 (95 residues), elafin (57 residues) and SLPI (107 amino acids) all have structurally homologous WAP (whey acidic protein) domains that each contain four disulphide bonds, and are responsible for their inhibitory activity. Trappin-2 and elafin each contain one WAP domain while SLPI has two. The N-terminal part of trappin-2 or cementoin domain [4] has a unique cysteine-free sequence of 38 residues containing five repeated motifs conforming to the consensus sequence GQDPVK and thought to be transglutaminase substrates [4]. This finding led to the hypothesis that trappin-2 could be covalently anchored at its site of action, probably by transglutaminase-catalysed cross-linking to extracellular matrix proteins [4]. Indeed, transglutaminase-2 (TGase), which is ubiquitously expressed in cells and is secreted into the extracellular space [5], mediates the cross-linking of trappin-2 to human epidermis proteins both *in vitro* and *in vivo* [6]. Other *in vitro* studies have reported that trappin-2 is an efficient TGase substrate, as it can be cross-linked to laminin [4], elastin [7], fibronectin and other extracellular matrix (ECM) proteins [8].

Trappin-2 and/or elafin have also been detected in the trachea mucous epithelium by Western blotting. But their apparent Mr was much higher (∼50 kDa) than expected (∼6–12 kDa), suggesting that they were cross-linked to ECM proteins [4]. Another study by Steinert and Marekov [9] identified several proteins from the human stratum corneum that were cross-linked by transglutaminases, including trappin-2 and to a lesser extent elafin. They were mostly conjugated to loricrin, accounting for about 6% of all the protein components of the cornified cell envelope. Besides this structural role of trappin-2/elafin engaged in protein-protein cross-links with various ECM proteins, the biological role of conjugated inhibitors is not yet clear. The covalent attachment of trappin-2 to protease-sensitive proteins could be essential for protecting them from proteolytic degradation by neutrophil elastase or proteinase 3, both inhibited by trappin-2 and elafin. Recent research in our laboratory has shown that trappin-2 conjugated to fibronectin by transglutaminase-2 (TGase) is almost as active a protease inhibitor as soluble trappin-2 [8], thus confirming the defence-assistance role of anchored trappin-2 originally proposed by Nara et al. [4]. This capacity of trappin-2 and its genetically modified variants [10] to become immobilized on ECM proteins in an interesting property in the context of their possible use as aerosol-delivered anti-inflammatory therapeutic agents for treating lung diseases. Such cross-linked inhibitors could have longers half-lives at inflammatory sites than the soluble proteins.

SLPI does not have any consensus transglutaminase substrate sequence(s) but being homologous to trappin-2/elafin, we investigated whether it could be a transglutaminase substrate. We have now demonstrated that SLPI is readily cross-linked to fibronectin and elastin by tissue transglutaminase-2 and plasma factor XIIIa. And the covalently bound SLPI retains its ability to inhibit neutrophil elastase (HNE) and cathepsin G (CatG). Further, to gain mechanistic insight into the sequence and structural determinants that allow transglutaminases to cross-link these inhibitors, we used mass spectrometry to identified a set of reactive glutamine and lysine residues in trappin-2, elafin and SLPI that are targeted by transglutaminase-2.

## Results

### TGase- and factor XIIIa-directed incorporation of dansyl-cadaverine and peptide substrates into elafin, trappin-2 and SLPI

**Figure 1:**
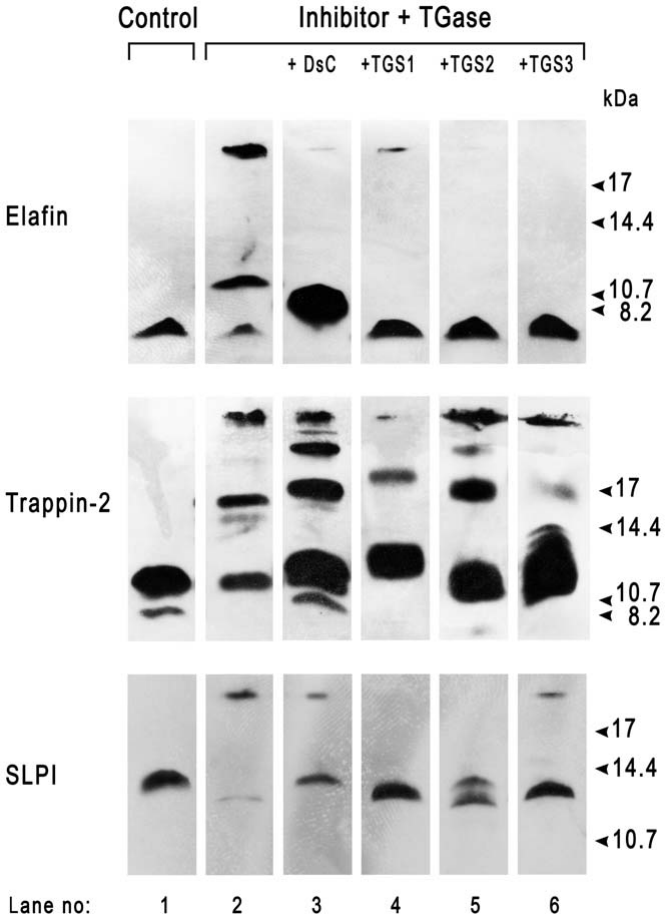
TGase-mediated incorporation of DsC and peptides substrates in elafin, trappin-2 and SLPI. Each inhibitor (1.2×10^−5^ M) was incubated with TGase (1.25×10^−7^ M) for 1 h at 37°C either alone (lane #2), with dansyl-cadaverin (DsC) (2 mM), or with the glutamine-containing peptides TGS1, TGS2 and TGS3 (2.4 mM each). Products were separated by high resolution 16% SDS-PAGE electrophoresis and detected by Western blotting using primary antibodies specific for each inhibitor and peroxidase-labelled secondary antibodies. The right-hand lane in each panel contains molecular mass standards.

We performed transglutamination assays to determine whether the lysine or glutamine residues in elafin and trappin-2 are the primary targets in transamidation reactions catalyzed by TGase or FXIIIa. We used labelled primary amine dansyl-cadaverin (DsC), which is widely used in transamidation assays because it mimics a lysine side-chain, or three glutamine-containing peptides. The peptide TGS1 with the sequence PGGQQIV is derived from fibronectin [11] while TGS2 (HQSYVDPWMLDH) and TGS3 (DQMMLPWPAVAL) have been shown by phage display to be efficient transglutaminase substrates; TGS2 is selective for TGase and TGS3 for FXIIIa [12]. Elafin readily formed complexes with molecular weights of about 12 kDa or >17 kDa, as assessed by Western blotting, in the presence of TGase (Fig. 1). Thus elafin is a transglutaminase substrate for itself, with cross-linking through isopeptide bonds formed between two or more elafin molecules. Adding DsC or glutamine-containing peptides (TGS1, TGS2, TGS3) to the incubation mixture almost totally inhibited the formation of elafin-elafin complexes, even with TGS3, which is supposed to be a poor substrate for TGase. This indicates that elafin contains both reactive lysine and glutamine residues that participate in the formation of isopeptide bonds and that these residues of elafin are less efficient TGase substrates than are DsC, TGS1, TGS2 or TGS3. Hence, TGase targets these small molecules rather than elafin in the transamidation reaction.

We observed similar results with trappin-2 as a TGase substrate. Cross-linking between trappin-2 molecules was inhibited by TGS1, TGS2 and TGS3 peptides. But, in contrast to elafin, inhibition was not total, suggesting that the additional Lys residues in the cementoin domain of trappin-2 also served as TGase substrates even in the presence of peptide substrates. Complex formation was also inhibited by DsC (Fig. 1). However, the formation of complexes with different molecular weights indicates that cross-linking occurred through alternative transglutamination sites when DsC was included in the reaction mixture. As TGS2 and TGS3 have been reported to be poor glutamine donor substrates when reacted with the trappin-2-derived peptide GQDPVK [12], we believe that other Lys residues in elafin or trappin-2, including that of the AQEPVK motif at the N-terminus of elafin, serve as acyl acceptors in transamidation reactions involving elafin or trappin-2.

We next determined whether SLPI was a transglutaminase substrate *in vitro*. Unlike trappin-2, SLPI does not contain motifs containing the consensus sequence GQDPVK that has been reported to be a substrate for tissue transglutaminases [13]. However, the N-terminal domain of SLPI contains a similar motif having the W_30_QCPGK_35_ sequence, whose Gln and/or Lys residues could be a TGase target. SLPI readily formed complexes larger than 17 kDa in the presence of TGase, as indicated by SDS-PAGE analysis (Fig. 1). This suggests that TGase catalysed the cross-linking of two SLPI molecules. We confirmed the presence of reactive glutamine and lysine residues in SLPI by incubating SLPI with DsC or glutamine-containing peptides. As with elafin and trappin-2, DsC and the peptides TGS1, TGS2, TGS3 all inhibited the TGase-catalysed formation of SLPI complexes. Thus SLPI is a TGase substrate for TGase and contains both reactive Gln and Lys residues that can cross-link SLPI, DsC or Gln-containing peptides.

**Figure 2:**
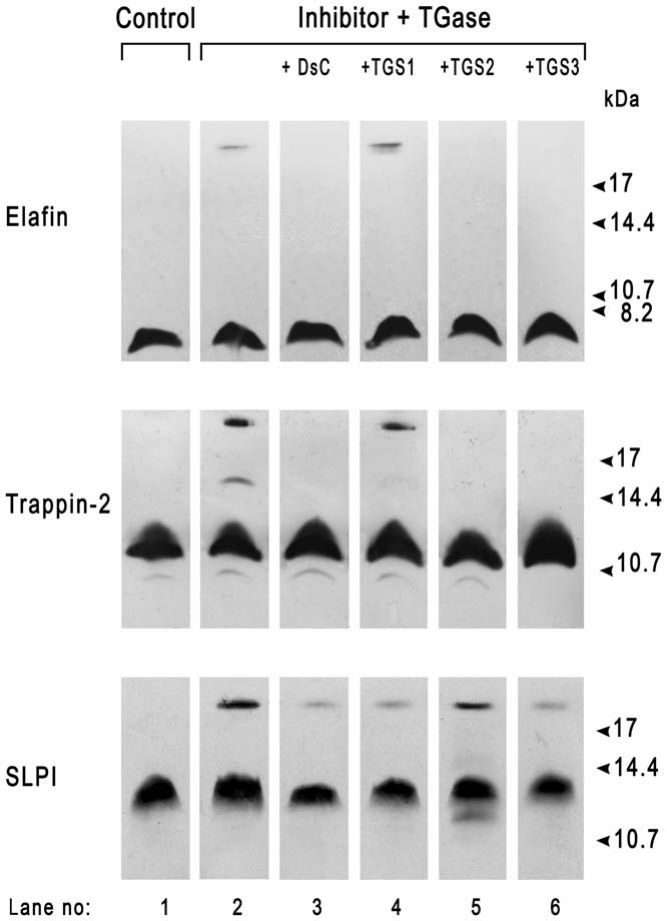
FXIIIa-catalysed incorporation of DsC and peptides substrates in elafin, trappin-2 and SLPI. Conditions were the same as in Fig. 1 except that FXIIIa was the transglutaminase.

We repeated these experiments on elafin, trappin-2 and SLPI with FXIIIa as the transglutaminase, to determine the effects of DsC and Gln-containing peptides. Elafin and trappin-2 may be substrates of FXIIIa *in vitro* since they both formed high molecular weight complexes when incubated with this enzyme (Fig. 2), as we observed previously [8]. Elafin formed only one additional molecular form, in contrast to its reaction with TGase, perhaps because it has fewer transglutamination sites for FXIIIa. DsC, TGS2 and TGS3 inhibited the transglutamination of elafin and trappin-2 to themselves, but TGS1 did not. This indicates that TGS1 is not a substrate for FXIIIa and that TGS2, which has been reported to be selective for TGase [12], may also be an FXIIIa substrate. This also confirms that elafin and trappin-2 contain both reactive Gln and Lys residues that are substrates for tranglutaminases. FXIIIa catalysed the cross-linking of SLPI to form complexes >17 kDa, and this complex formation was slightly inhibited by DsC, TGS1 and TGS3 but not by TGS2.

### Cross-linking of trappin-2 and SLPI to different target residues on fibronectin and elastin

**Figure 3:**
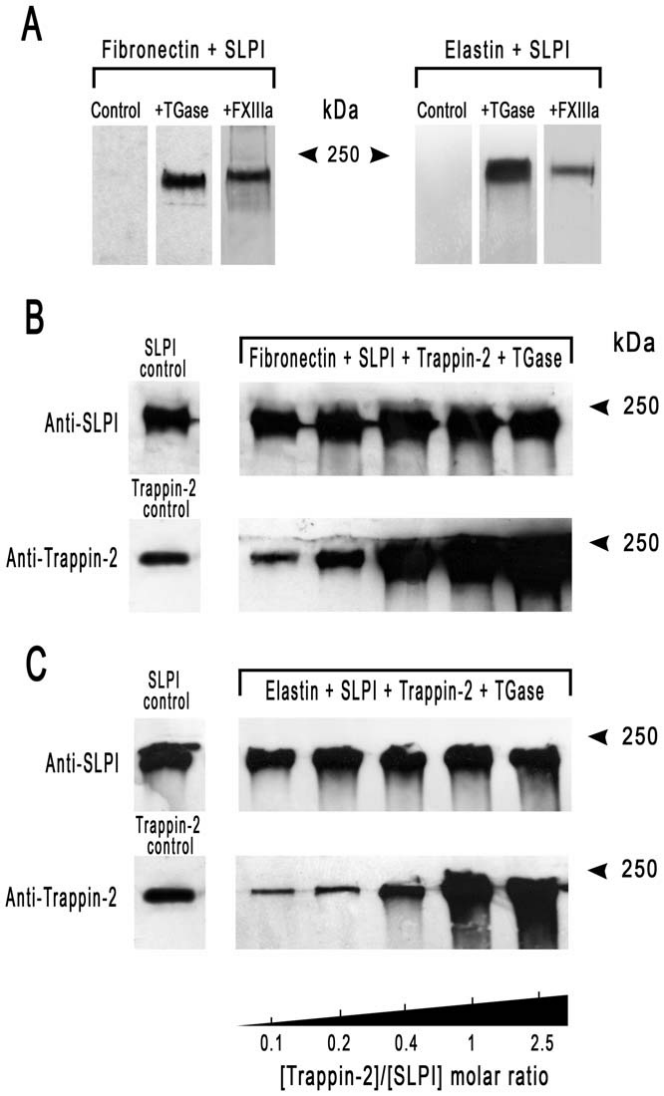
Influence of trappin-2 on the transglutamination-mediated binding of SLPI to fibronectin and elastin. (A) SLPI was cross-linked to fibronectin or to elastin by TGase or by FXIIIa. SLPI (3×10^−6^ M) was incubated with fibronectin (3 µg) or elastin (5 µg) plus TGase (1.25×10^−7^ M) or FXIIIa (10^−6^ M) for 2 h at 37°C. Cross-linked products were separated by SDS-PAGE in 10% Tris-Glycine and detected by Western blotting with anti-SLPI antibodies. (B, C) Competition between SLPI and trappin-2 for their transglutaminase-catalysed incorporation into fibronectin (B) or elastin (C). SLPI (3×10^−6^ M) was incubated for 2 h at 37°C with trappin-2 (3×10^−7^ to 7×10^−6^ M), fibronectin (3 µg) or elastin (5 µg) and TGase (1.25×10^−7^ M). The products were separated in parallel runs on two 10% SDS-PAGE gels and detected by Western blotting using anti-SLPI antibodies for the first gel and anti-trappin-2 antibodies for the second gel. The SLPI control and trappin-2 control refer to the TGase-catalysed cross-linking of SLPI and trappin-2 alone to either fibronectin or elastin. Similar results were obtained using FXIIIa (not shown) indicating that the transglutaminase-catalysed conjugation of SLPI and trappin-2 to either fibronectin or elastin do not compete; each inhibitor has its own transglutamination sites on these extracellular matrix proteins.

We and others have demonstrated that elafin and trappin-2 can be covalently bound to fibronectin, elastin and other extracellular matrix proteins in a reaction catalysed by a tissue transglutaminase. Although SLPI is similar to elafin and trappin-2, there have been no published reports of its capacity to be a transglutaminase substrate. However, it does become associated with elastin fibers *in vivo* by a yet unknown mechanism [14], [15], which could involve transglutamination. We assessed the transglutamination of SLPI by incubating SLPI with fibronectin or elastin plus the enzymes TGase or FXIIIa. SLPI readily formed high-molecular weight complexes of about 250 kDa with both fibronectin and elastin (Fig. 3). Since elafin/trappin-2 and SLPI can be transglutaminated to the same substrates, we next performed transglutamination assays in which SLPI and trappin-2 competed for TGase-catalysed binding to fibronectin or elastin. The SLPI concentration was kept constant and the trappin-2 concentrations were increased in a reaction mixture containing fibronectin or elastin plus TGase. Western blotting analysis indicated that the binding of SLPI to fibronectin or elastin remained unchanged while the formation of complexes involving trappin-2 increased with the trappin-2 concentration (Fig. 3) up to a 2.5 fold molar excess of trappin-2 over SLPI. Similar results were obtained using FXIIIa as a transglutaminase (not shown). Thus trappin-2 and SLPI are not mutually exclusive in their transglutaminase-mediated binding to extra cellular matrix proteins.

### Protease inhibitory activity of SLPI cross-linked to fibronectin or elastin by transglutamination

**Figure 4:**
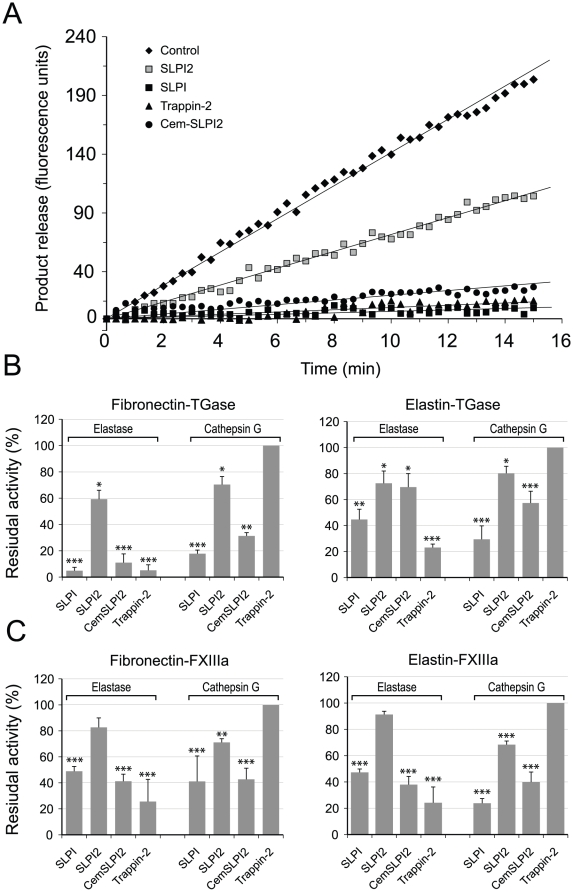
Inhibition of neutrophil elastase and cathepsin G by inhibitors bound to fibronectin or elastin. Inhibitors (10^−6^ M) (SLPI, SLPI domain 2 (SLPI2), trappin-2 and Cem-SLPI2, a fusion protein containing SLPI2 with the cementoin domain of trappin-2 fused to it N-terminus) were first cross-linked to fibronectin or elastin in 96-well microplates by incubation with TGase or FXIIIa for 2 h at 37°C. The microplate wells were then washed thoroughly to remove unreacted products and incubated with HNE (1 nM) or cathepsin G (2 nM) for 15 min at 37°C to form the protease-inhibitor complexes. A fluorogenic substrate specific for each protease was then added to the wells to measure the residual enzyme activity. (A) Inhibition of HNE by inhibitors bound to fibronectin by TGase (product release expressed as fluorescence units/time). (B, C) Residual HNE or cathepsin G activities after incubation with inhibitors conjugated to fibronectin or elastin by TGase (B) or FXIIIa (C), calculated as the ratio of substrate hydrolysis rates with and without inhibitor (Control). Data are represented as means ± SEM (n = 6) of residual enzymatic activity and were compared to the control (***p≤0.001, **p≤0.005 and *p≤0.05) using the post hoc student test.

We have shown that elafin and trappin-2 covalently bound to fibronectin by TGase are still active protease inhibitors [8], although they associate with their target proteases (HNE and Pr3) more slowly than do the soluble inhibitors, probably because of their immobilization. Because both the elafin-like domains of SLPI contain many Gln and Lys residues that are potential transglutaminase targets, the question arose whether cross-linking of SLPI onto fibronectin or elastin affects its inhibitory properties, bearing in mind that only SLPI domain 2 (SLPI2) inhibits the target neutrophil proteases, HNE and CatG [16], [17]. Fibronectin or elastin was first adsorbed onto high-adsorption ELISA plates and then SLPI was conjugated by TGase. We then used the same enzyme-based assay as that previously described [8] to measure the residual proteolytic activity of HNE or CatG in the presence of SLPI conjugated by TGase to immobilized fibronectin or elastin. We postulated that transglutamination involved SLPI1 rather than SLPI2 because SLPI domain 1 (SLPI1) has a sequence motif W_30_QCPGK_35_ that is similar to the GQDPVK transglutaminase substrate motif (*vide ante*) and because it has more Gln and Lys residues (3 Gln, 9 Lys) than does SLPI2 (2 Gln, 6 Lys). We tested this hypothesis by comparing the inhibitory properties of cross-linked full-length SLPI with those of cross-linked SLPI2 and Cem-SLPI2, a protein in which SLPI2 is fused at its N-terminal moiety to the cementoin domain of trappin-2, which has been reported to contain the main transglutamination sites in trappin-2 [4], [13]. Full-length SLPI was still an active inhibitor when bound to fibronectin and its capacity to inhibit HNE was about 95% of the control (Fig. 4). SLPI was less active (about 60%) when it was conjugated to elastin. TGase-conjugated trappin-2 behaved similarly, suggesting that elastin is a poorer TGase substrate than is fibronectin, as already mentioned for peptides TGS1, TGS2 and TGS3. Cross-linked SLPI2 was a far less potent inhibitor (≈40% inhibition) than SLPI, while Cem-SLPI2 conjugated to fibronectin appeared to be a potent inhibitor of HNE (≈90%). The same tendency was observed using CatG as a target enzyme except that immobilized trappin-2 did not inhibit CatG as expected (Fig. 4). Because SLPI2 and Cem-SLPI2 have the same inhibitory potency as SLPI (*K*_i_ = 5×10^−11^ M for SLPI2-HNE, 2.6×10^−10^ M for SLPI2-CatG, 2.7×10^−11^ M for Cem-SLPI2/HNE, 7×10^−10^ M for Cem-SLPI2-CatG), differences in inhibitory activities of cross-linked inhibitors are mainly related to the efficiency of the transglutamination reaction in which they are involved. Inhibitors cross-linked by FXIIIa appeared to be less active inhibitors, probably as a consequence of being less efficiently transglutaminated by FXIIIa compared to TGase, using similar enzyme concentrations. Thus, these results support our hypothesis that most of the transglutamination sites of SLPI are located in SLPI1 rather than in the inhibitory SLPI2 domain. We have also shown that the cementoin domain of trappin-2 can almost fully or partially restore, depending on the substrate, the transglutamination potential of SLPI when fused to SLPI2.

### Identification of TGase-reactive glutamine and lysine residues in elafin, trappin-2 and SLPI by mass spectrometry

We used two approaches to identify the reactive glutamine and lysine residue(s) within each inhibitor that reacted with TGase. In the first, we labelled each inhibitor with either DsC or the fibronectin-derived peptide PGGQQIV (TGS1) using TGase and then performed MALDI-TOF mass spectrometry analysis of crude reaction products or of fragments produced by trypsin digestion of cross-linked products. MALDI-TOF analysis of the non-digested reaction products (not shown) revealed that SLPI and trappin-2 were substituted by either one, two or three DsC or TGS1 peptides. In the SLPI-TGS1-TGase mixture, we also observed a fourth form which most probably contained four TGS1-derived Lys. With elafin reacting with either DsC or TGS1, we observed one major peak in the MALDI-TOF spectrum suggesting that the mono-substituted form of elafin was likely to be predominant in the reaction mixture. All these molecular forms were already observed after 5 min. incubation with TGase and progressively increased as a function of time while the native form decreased at the same time. But no additional m/z signals appeared up to 1 h incubation with TGase. Since MALDI-TOF analysis do not allow accurate quantitations, we did not attempt to determine the exact proportion of each form. Altogether these results suggest that each inhibitor is present in several molecular forms reflecting the fact that a restricted, preferential set of Gln or Lys residues are targeted by TGase.

**Figure 5:**
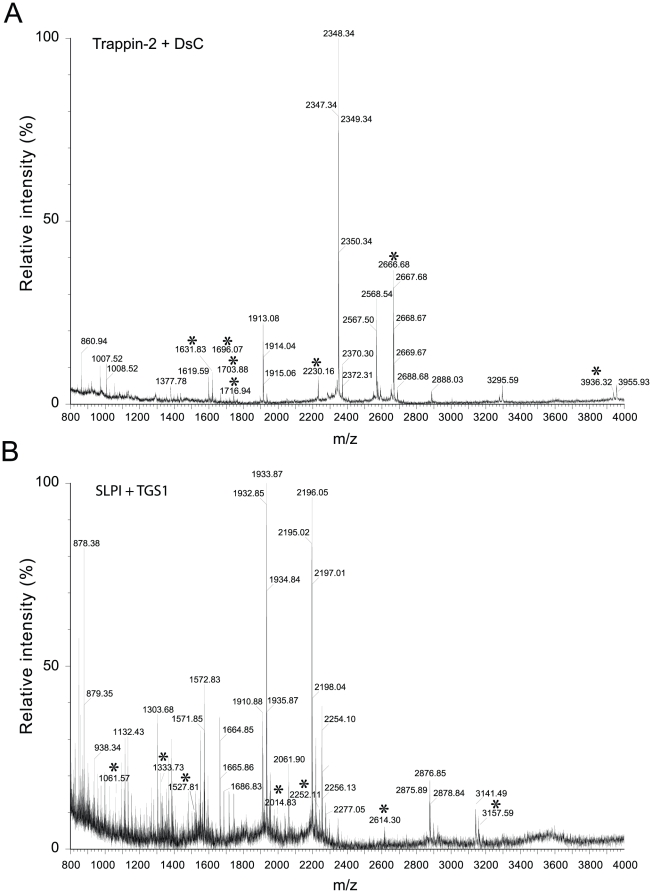
MALDI-TOF mass spectra of peptides generated from a trypsin digest of TGase-modified inhibitors. A) Trappin-2 was incubated with DsC plus TGase for 2 h at 37°C, reduced and alkylated, and digested with trypsin overnight. B) SLPI was labelled with the TGS1 peptide by TGase and then hydrolysed with trypsin. Those tryptic peptides that were not present in the control digest, and consequently probably contained amino acids modified by TGase activity, are indicated by an asterisk. The experimental masses of these peptides were compared with the masses of theoretical tryptic peptides to identify modified amino-acids (Table 1).

**Figure 6:**
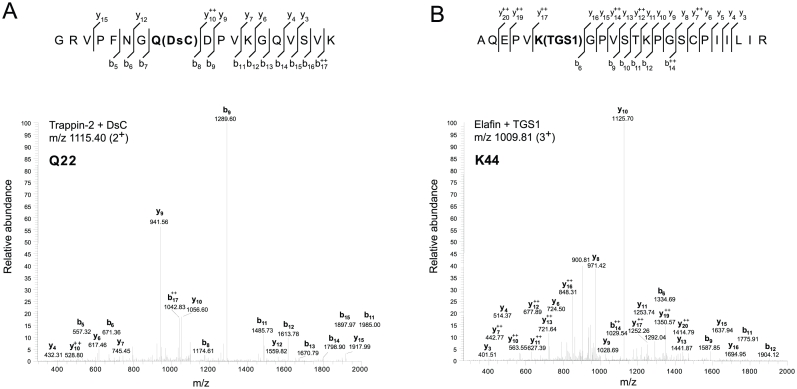
MS-MS mass spectrometry analysis of the tryptic peptides generated from DsC- or TGS1-labeled inhibitors. Certain tryptic peptides were further fragmentated and analyzed by MS-MS to unambiguously identify the glutamine or lysine residues in trappin-2, elafin or SLPI targeted by TGase. Modified glutamine and lysine were identified based on their altered molecular mass due to Dsc and TGS1 cross-linking by TGase respectively, while amino acid sequences were obtained from the peptide fragmentation patterns. The figure shows two representative MS-MS spectra, one for a peptide with a m/z of 1115.40 (2^+^) from the tryptic digest of DsC-labelled trappin-2 (A) and the second with a m/z of 1009.81 (3^+^) found after tryptic hydrolysis of elafin cross-linked to TGS1 peptide. The fragmentation pattern with experimentally-assigned ions is shown above each spectrum. For the sake of clarity, not all peaks of daughter ions were labelled on each spectrum.

**Figure 7:**
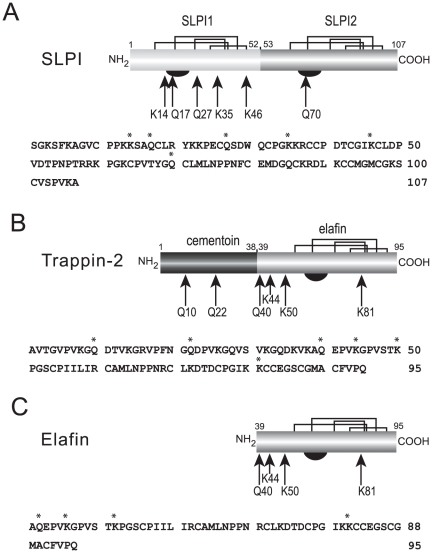
Location of TGase-reactive Gln and Lys residues in SLPI, trappin-2 and elafin. Structural organization of SLPI (A) with its two elafin-like domains, trappin-2 (B) and elafin (C). The amino acid sequence of each inhibitor is shown below each structure. The disulfide bond topology (plain lines) and the inhibitory loop (half black disc) of each inhibitor are also shown. Arrows show the TGase-reactive Gln residues in SLPI (17, 27, 70) and trappin-2 (10, 22, 40) and the reactive Lys residues in SLPI (14, 35, 46) and trappin-2 (44, 50, 81). The reactive residues in elafin (trappin-2 numbering) were the same as those in the elafin moiety of trappin-2. Asterisks above the amino acid sequence denote the location of the reactive residues in each inhibitor.

In order to identify these TGase-sensitive residues, we then performed tryptic peptide map MALDI-TOF analysis (Fig. 5). Because these cationic inhibitors contain many Arg and Lys residues, the tryptic fragments obtained were small enough for direct analysis. The MS data were matched automatically to a database containing the trappin-2, elafin and SLPI protein sequences using the Peptide Mass Fingerprint search option of the MASCOT server software. Sequence coverage for MS analysis of DsC-derived fragments was 100% for trappin-2 and elafin, 90% for SLPI; it was 92% for trappin-2 fragments modified by TGS1, 100% for elafin fragments and 96% for SLPI fragments. Reactive glutamine and lysine residues were identified from the altered mass of the fragments containing DsC-derived glutamine (+318 Da) or TGS1-lysine (+681.38 Da) and the release of an ammonia molecule (17.03 Da) for each ε-(γ-glutamyl)-lysine cross-link formed by TGase catalysis (Table 1). Some tryptic fragments containing two or three lysines (e.g fragment 70–95 in elafin) were substituted by one or more TGS1 peptides so that non-ambiguous identification of reactive lysines by inference was not possible in these conditions. These peptides were further fragmentated and analyzed by nanospray LC-MS/MS (Fig. 6). Peptides were considered to be identified with certainty when five consecutive fragment ions were obtained in the MS/MS analysis. Our data (Fig. 7) indicate that Lys44 is the preferred reactive lysine in trappin-2 and elafin, while Lys50 and Lys81 may also be targeted by TGase for TGS1 cross-linking. Lys72 or Lys80, which MALDI-TOF data suggested were conjugated to TGS1 (Table 1), were not unambiguously confirmed by MS/MS. The MS/MS data obtained for SLPI indicated that three lysines (Lys14, Lys35 and Lys46) were TGase substrates for TGS1 conjugation (Fig. 7). Lys46 was inferred from MS/MS fragmentation of the Cys38-Arg58 SLPI fragment, although this latter was not retrieved by the automatic detection of mass peaks in the MALDI-TOF analysis (Table 1). On the other hand, several putative reactive lysines in SLPI (Table 1) were not confirmed by MS/MS analysis and were considered to be artefacts due to the moderate robustness of the MALDI-TOF-derived sequence assignment compared to MS/MS analysis. All assignments of reactive glutamines from MALDI-TOF data for the three inhibitors were confirmed by MS/MS analysis of DsC-conjugated peptides. The MS/MS analysis of some peptides not detected as Dsc-derived in MALDI-TOF analysis also enabled us to identify two other reactive glutamines in SLPI, Gln27 and Gln70. They were identified unambiguously from the fragmentation of Tyr21-Lys35 and semi-tryptic Cys64-Leu72 and Arg59-Leu72 SLPI peptides. Locations of TGase-sensitive residues in all three inhibitors are shown in Fig. 7.

**Table 1:** MALDI-TOF mass spectrometry analysis of tryptic peptides generated from dansylcadaverin- and PGGQQIV-labeled trappin-2, elafin and SLPI.

		[M+H]^+^			
Peptide fragment	Amino acid sequence	observed	theoretical	Δmass	Substitution
	**Trappin-2 + DsC**				
1–14	AVTGVPVKGQDTVK	1716.94	1398.79	318.14	DsC-Q10
15–26	GRVPFNGQDPVK	1631.83	1313.69	318.13	DsC-Q22
15–32	GRVPFNGQDPVKGQVSVK	2230.19	1912.03	318.15	DsC-Q22 or Dsc-Q28
39–60	AQEPVKGPVSTKPGSCPIILIR	2665.78	2347.31*	318.46	DsC-Q40
	**Elafin + DsC**				
39–60	AQEPVKGPVSTKPGSCPIILIR	2665.68	2347.31*	318.36	DsC-Q40
	**SLPI + DsC**				
7–20	AGVCPPKKSAQCLR	1889.99	1571.81*	318.17	DsC-Q17
14–20	KSAQCLR	1180.61	862.45*	318.16	DsC-Q17
	**Trappin-2 + TGS1**				
39–60	AQEPVKGPVSTKPGSCPIILIR	3028.51	2347.31*	681.19	TGS1-K44 or TGS1-K50
73–80	DTDCPGIK	1528.77	848.38	680.39	TGS1-K80
	**Elafin + TGS1**				
39–60¶	AQEPVKGPVSTKPGSCPIILIR	3028.56	2347.31*	681.24	TGS1-K44 or TGS1-K50
70–95	CLKDTDCPGIKKCCEGSCGMACFVPQ	4440.48	3078.27*	1362.20	2 substitutions among TGS1-K72, TGS1-K80, TGS1-K81
73–81	DTDCPGIKK	1713.92	1033.49	680.42	TGS1-K80 or TGS1-K81
	**SLPI + TGS1**				
1–6	SGKSFK	1333.73	653.36	680.37	TGS1-K3 or TGS1-K6
4–6	SFK	1061.56	381.21	680.35	TGS1-K6
4–20	SFKAGVCPPKKSAQCLR	2614.30	1934.00	680.29	TGS1-K6 or TGS1-K13 or TGS1-K14
7–14	AGVCPPKK	2217.05	856.47	1360.58	TGS1-K13 and TGS1-K14
7–20	AGVCPPKKSAQCLR	2252.11	1571.81*	680.29	TGS1-K13 or TG1S-K14
7–20	AGVCPPKKSAQCLR	2253.17	1571.81*	681.36	TGS1-K13 or TG1S-K14 + deamidation of Q17
7–20	AGVCPPKKSAQCLR	2875.80	1514.79*	1361.01	TGS1-K13 and TGS1-K14
21–35	YKKPECQSDWQCPGK	3157.59	1796.80	1360.78	2 substitutions among TGS1-K22, TGS1-K23, TGS1-K35
38–58	CCPDTCGIKCLDPVDTPNPTR	3157.59	2476.06*	681.52	TGS1-K46
89–99	DLKCCMGMCGK	2014.83	1334.50*	680.33	TGS1-K91 or TGS1-K9 (oxidized methionins)
100–107	SCVSPVKA	1527.81	847.43*	680.37	TGS1-K106

Each labeled inhibitor was hydrolysed with trypsin, the resulting peptides reduced with DTT and alkylated with iodoacetamide before MALDI-TOF analysis. Putative glutamine and lysine residues targeted by TGase were identified by the altered mass of corresponding tryptic peptides bearing DsC on Gln residues or the PGGQQIV peptide (TGS1) on Lys residues. The release of an ammonia molecule that accompanies the formation of an isopeptide bond by TGase indicated that the expected increase in mass of DsC-labeled Gln is +318 Da (335-17) and that of TGS1-labeled Lys is +681.38 Da (698.38-17). The theoretical masses of unsubstituted tryptic peptides were calculated with PeptideMass software (http://www.expasy.ch/tools/peptide-mass.html).

*theoretical mass calculated assuming carbamidomethyl-Cys (carboxyamido 57.0214 g/mol).

¶ trappin-2 numbering.

## Discussion

We previously showed that elafin and trappin-2 cross-linked to various ECM proteins by the catalytic action of a tissue transglutaminase retain their capacity to inhibit serine proteases [8]. We have now demonstrated that the homologous SLPI molecule can also be cross-linked to fibronectin and elastin by tissue transglutaminase, despite its lack of the repeated consensus sequence transglutaminase motif Gly-Gln-Asp-Pro-Val-Lys (GQDPVK) found in trappin-2 [13]. We have also shown that both elafin/trappin-2 and SLPI are targeted by FXIIIa. To our knowledge, this is the first report of the transglutamination of SLPI. It was readily cross-linked to itself or to fibronectin and elastin by two different transglutaminases. Western blotting analysis indicated that a Gln-containing peptide (TGS1) based on the N-terminal sequence of fibronectin [18] and Lys-containing peptides identified by phage display [12] that are preferred substrates of TGase (TGS2) or FXIIIa (TGS3), interfered with the transglutamination patterns of all three inhibitors, elafin, trappin-2, and SLPI. This suggests that these inhibitors contain reactive lysine and glutamine residues that are transglutaminase targets. Because both trappin-2 and SLPI may be cross-linked to fibronectin or elastin, we set out to answer the question of whether these inhibitors occupied the same transglutamination sites on these ECM proteins. The transglutaminase-catalyzed cross-linking of SLPI to fibronectin or elastin was not modified by excess trappin-2, while trappin-2 conjugation increased as the trappin-2/SLPI molar ratio increased. This clearly indicates that SLPI and trappin-2 do not compete for the same binding sites. Although fibronectin has a restricted number of glutamine residues that can be transglutaminated by TGase or FXIIIa [19], the binding of the two inhibitors is not mutually exclusive.

We first tried to identify the reactive glutamine and lysine residues involved in the cross-linking of inhibitors to fibronectin by mass spectrometry analysis of tryptic digests of fibronectin-trappin-2 conjugates in order to infer the positions of reactive lysines or glutamines from the masses of tryptic fragments linked by isopeptide bonds. But we could not accurately correlate the experimental masses with the sequences of either the inhibitor or fibronectin, probably because our commercial fibronectin was not pure enough and/or there are numerous isoforms of human fibronectin, so no unique sequence is present. Instead, we carried out analysis by mass spectrometry of tryptic digests of TGase-catalyzed cross-linked products involving each inhibitor and either DsC as a lysine mimic or the glutamine-containing peptide TGS1 as transglutamination probes. MALDI-TOF analysis allowed us to deduce most of the TGase-reactive lysine and glutamine residues in SLPI, elafin and trappin-2 from the masses of tryptic peptides. When tryptic peptides of inhibitors contained a number of potentially reactive residues >1, we used MS/MS analysis to identify unambiguously cross-linked lysine or glutamine residues within each inhibitor. We found that three of the 5 glutamines in SLPI, Gln17, Gln27 and Gln70, were amine acceptors, while three of the 6 potential glutamines in trappin-2, Gln10, Gln22, and Gln40, were targeted in trappin-2. Labeling with DsC revealed that 3 of the 15 lysines in SLPI, Lys14, Lys 35 and Lys 46, had incorporated the probe while the 11 lysines in trappin-2 included three, Lys 44, Lys 50 and Lys 81, that could serve as TGase substrates. Compared to trappin-2, no difference in TGase reactivity of elafin alone was observed which had the same reactive Lys and Gln residues as those identified in the elafin moiety contained in trappin-2. The five GQDPVK sequence motifs of trappin-2 were previously found to be similar to the seminal vesicle secretory protein SVP-1, involucrin and cornifin, all of which are transglutaminase substrates [4]. This led to the early hypothesis that these motifs lying mostly in the N-terminal cementoin domain were responsible for the covalent anchoring of the elastase inhibitor trappin-2/elafin at its site of action by transglutamination [4]. The only data on the transglutamination sites in elafin/trappin-2 came from a study of proteins forming the human epidermal cornified cell envelope. Trappin-2 was found in transglutaminase-catalyzed cross-links with the epidermal proteins loricrin, desmoplakin, involucrin and keratin I [9]. Identification of transglutamination sites from the amino acid composition studies of proteinase K digests of stratum corneum extracts revealed that most cross-links with trappin-2 involved its Gln40 and Lys44 residues, but Lys36, Lys38 and the C-terminal Gln95 were also minor sites. We also found Gln40 and Lys44 to be major sites, but we found no cross-links with Lys36, Lys38 or Gln95. This suggests that the use of alternative reactive Lys or Gln residues may depend on the type of transglutaminase and/or the second substrate involved in the transamidation reaction. Surprisingly no Gln or Lys residues contained in the GQDPVK motifs within the cementoin domain were found to be reactive with epidermal proteins, although these motifs were initially identified as transglutaminase substrate sequences in trappin-2 [4]. Furthermore, those lysines in the cementoin domain that are in the same sequence context as Lys44 (i.e included in a PVKG sequence) do not seem to react with TGase. This implies that TGase is remarkably specific for certain Gln and Lys sites in both trappin-2/elafin and SLPI, as it is for many other proteins [12], [20], [21]. Our results provide no clear consensus sequence around reactive Gln or Lys residues. Of the six Gln targeted in trappin-2/elafin and SLPI, two of them were found in a QXPΦ motif (where X is any amino acid and Φ a hydrophobic amino acid). This motif appears to be preferred by the tissue transglutaminase 2 used in our study [12]. Three other reactive Gln were included in a QXXΦ motif, suggesting that a hydrophobic residue at +3 is important for determining substrate selectivity. There was the same lack of a consensus sequence around the reactive Lys residues in both inhibitors. But the Lys35 in SLPI lies within a WQCPGK sequence that resembles the proposed GQDPVK consensus transglutaminase substrate sequence of the cementoin domain of trappin-2. We were also unable to establish any correlation between the reactivity of the Gln and Lys residues and their accessibilty in the 3D structure of SLPI and the elafin moiety of trappin-2 (data not shown), in agreement with previous findings suggesting that other structural determinants in the vicinity of reactive residues are important [20], [21].

Because trappin-2 appeared to be a better TGase substrate than SLPI with all substrates tested, including involucrin (data not shown), we examined the capacities of the two inhibitors to compete in the transglutaminase-catalysed binding to various ECM proteins. Experiments with fibronectin, which has eight TGase binding sites per intact molecule (four per 220 kDa monomer) [19], showed that SLPI and trappin-2 were not competing with each other; the same was true for binding to elastin. Our findings that SLPI may be cross-linked to elastin by a transglutaminase-mediated process may explain why this inhibitor was found to be associated with elastin fibers in the skin [15] and in the parenchymal matrix of the lung alveolar walls [14] by a hitherto unknown mechanism. We do not know whether trappin-2 is also specifically conjugated to elastin *in vivo*, although there is evidence that it can be detected as high-molecular weight forms in psoriasis skin or lung tissues, as a probable consequence of tranglutaminase-mediated conjugation [4], [6]. As previously suggested [4], [7], [8], the obvious biological function of the transglutaminase-catalysed covalent linking of trappin-2/elafin to ECM proteins is to protect them from degradation by neutrophil serine proteases, so helping maintain tissue integrity during inflammation and/or tissue remodelling. We have shown that SLPI covalently bound to fibronectin or elastin also retains its ability to inhibit its target proteases (HNE and CatG). Hence, conjugated SLPI, together with trappin-2/elafin, may be important for preventing the breakdown of elastin and other structural proteins at inflammatory sites. Both SLPI and trappin-2 have anti-microbial activities (reviewed in [2]), but we do not yet know whether there is a link between their transglutamination and the recently described mechanism of pathogen entrapment by transglutaminase [22].

SLPI and trappin-2/elafin are not the only protease inhibitors targeted by transglutaminases. Three members of the cystatin superfamily of cysteine protease inhibitors, cystatin alpha [23], cystatin M/E [24] and cystatin CRES [25], two serpins, PAI-2 [26] and α2-antiplasmin [27], α2-macroglobulin [28] and *Streptomyces* subtilisin and TAMEP inhibitor (SSTI) [29] are all transglutaminase substrates. The major role of all these cross-linked inhibitors seems to be the regulation of protease activity, although it is always possible that these conjugated inhibitors could have other, so far unidentified, biological functions.

We have also shown that the fusion protein (Cem-SLPI2) containing the cementoin domain of trappin-2 linked to SLPI domain 2 is a far better transglutaminase substrate than is SLPI alone. This suggests that the cementoin domain has potential for biotechnological applications, such as the transglutaminase-catalysed immobilization of biologically active therapeutic proteins on fibronectin, elastin or solid supports, thereby increasing their bioavailability.

## Materials and Methods

### Materials

Human neutrophil elastase (HNE) (EC 3.4.21.37), was obtained from Biocentrum (Krakow, Poland), human proteinase 3 (Pr3) (EC 3.4.21.76) was from Athens Research and Technology (Athens, USA) and cathepsin G (CatG) (EC 3.4.21.20) was from MP Biomedicals (Vannes, France). Fluorogenic substrates specific for each neutrophil proteinase were custom-synthesized by Gencust Europe (Dudelange, Luxembourg). Human fibronectin, bovine neck elastin, dansylcadaverine (DsC) and guinea pig liver transglutaminase (TGase) (EC 2.3.2.13) were from Sigma-Aldrich (St Quentin Fallavier, France) and factor XIIIa was from Kordia (Leiden, The Netherlands). Transglutaminase peptide substrates PGGQQIV (TGS1), HQSYVDPWMLDH (TGS2) and DQMMLPWPAVAL (TGS3) were supplied by Genosphere Biotechnologies (Paris, France). Anti-SLPI antibodies were from Tebu-Bio (Le Perray en Yvelines, France). Rabbit anti-trappin-2 antibodies were prepared in house using recombinant trappin-2 as antigen. All other reagents were of analytical grade.

### Construction, expression and purification of SLPI, SLPI2 and Cem-SLPI2

Wild type SLPI, SLPI domain 2 (SLPI2) and Cem-SLPI2 were produced as tag-free recombinant proteins in the *Pichia pastoris* expression system by the procedure used to produce recombinant elafin and trappin-2 [30] using a cDNA encoding the full-length SLPI protein (a gift from Dr. M. Kemme, University of Darmstadt, Germany). The full length SLPI cDNA was amplified by PCR using *SLPIFor* (5′-CGACTCGAGAAAAGATCTGGAAAGTCCTTCAAAGC-3′) and *SLPI2rev* (5′-CGAGCGGCCGCGGAATCAAGCTTTCACAGG-3′) primers, and SLPI domain 2 (SLPI2) cDNA was amplified with *SLPI_2_For* (5′-CGACTCGAGAAAAGGGATCCTGTTGACACCCC-3′) and *SLPI2Rev* primers (MWG Biotech, Villebon sur Yvette, France).

Cem-SLPI2 cDNA was generated using the trappin-2 cDNA and the cDNA encoding the SLPI full-length protein as templates. The cementoin domain of trappin-2 was amplified using *CemFor* (CGACTCGAGAAAAGAGCTGTCACGGGAGTTCCT-3′) and *Rev* (5′-TGGGGTGTCAACAGGATCTTTGACTTTATCTTGACCTTTA-3′) as primers. The cDNA encoding the SLPI2 domain was amplified using For (5′-TAAAGGTCAAGTCAAAGATCCTGTGACCCCA-3′) and SLPI2Rev as primers. Full length chimera cDNA was obtained by fusing the two PCR products by the standard procedure of Higuchi *et al.* [31]. All the PCR reactions were done with Taq/Pwo DNA polymerase (Expand High Fidelity system) from Roche (Meylan, France). The cDNAs were cloned into the pPIC9 vector (Invitrogen, Groningen, The Netherlands) and electroporated into *Pichia pastoris* yeast strain GS115 (his4) competent cells (Invitrogen, Carlsbad, CA, USA). The inhibitors were then expressed and purified by cation exchange chromatography by the procedures used for elafin and trappin-2 [30]. The purified SLPI, SLPI2 and Cem-SLPI2 molecules migrated as single bands at 12 kDa, 6 kDa and 10 kDa in reducing SDS/PAGE gel, indicating their homogeneity.

### Transglutamination assays

#### TGase-/FXIIIa-mediated incorporation of DsC and peptides into elafin, trappin-2 and SLPI

Cross-linked complexes were formed by incubating elafin, trappin-2 and SLPI (each 1.2 10^−5^ M) with or without DsC (2 mM) or peptides TGS1, TGS2, TGS3 (2.4 mM) and TGase (1.25×10^−7^ M) or FXIIIa (2×10^−6^ M) in 50 mM Tris-HCl buffer, pH 7.5, 2 mM CaCl_2_, 0.1 mM dithiothreitol (DTT) for 1 h at 37°C. Samples were boiled and the peptides separated on high resolution Tris-Tricine SDS-PAGE gels [32] and the separated peptides transferred to nitrocellulose membranes (GE Healthcare, Orsay, France). Membrane free sites were blocked by incubation with PBS containing 5% skim milk for 1 h at room temperature. Elafin and trappin-2 were detected by incubation with our rabbit anti-trappin-2 (1/10000) antibodies and SLPI (1/2000) overnight at 4°C followed by appropriate HRP-coupled secondary antibodies (Sigma-Aldrich, St Quentin Fallavier, France). The washing buffer was PBS containing 1% Tween 20.

#### Competition for the TGase-/FXIIIa-catalysed cross-linking of SLPI and trappin-2 to the extracellular matrix proteins fibronectin and elastin

Fixed concentrations of SLPI (3×10^−6^ M) and increasing concentrations of trappin-2 (3×10^−7^ M to 7×10^−6^ M) were mixed with fibronectin (3 µg) or elastin (5 µg) and incubated with TGase (1.25×10^−7^ M) or FXIIIa (1×10^−6^ M) in 50 mM Tris-HCl buffer, pH 7.5, 2 mM CaCl_2_, 0.1 mM DTT for 2 h at 37°C. The samples were then boiled in denaturing buffer and the cross-linked complexes with fibronectin or elastin separated on 10% Tris-Glycine SDS-PAGE. The separated proteins were transferred to nitrocellulose membranes and the free sites on the membranes were blocked as described above. Trappin-2 was detected by incubation with our rabbit anti-trappin-2 (1/5000) antibodies, and anti-SLPI (1/1000) overnight at 4°C, followed by the appropriate HRP-coupled secondary antibodies (Sigma Aldrich, St Quentin Fallavier, France).

#### Kinetic studies with soluble neutrophil proteases

The equilibrium dissociation constant *K*_i_ for the interaction of SLPI2 and Cem-SLPI2 with HNE or CatG were determined by adding substrate to an equilibrium mixture of protease and inhibitor in 50 mM Hepes buffer, pH 7.4, 150 mM NaCl for HNE and in 50 mM Hepes buffer, pH 7.4, 50 mM NaCl for CatG at 37°C. Residual enzyme activities were measured using the specific fluorogenic substrates Abz-APEEIMRRQ-EDDnp for HNE and Abz-TPFSGQ-EDDnp for CatG (10 µM final). Substrate-independent *K*_i_ values were determined from experimental data as previously described [10].

#### Inhibitory properties of recombinant inhibitors cross-linked to fibronectin and elastin by tissue transglutaminase and factor XIIIa

ELISA microplates (96-well, Fluoronunc Maxisorp plates) were coated (2 µg/well) with fibronectin or elastin in 0.1 M sodium carbonate buffer (pH 9.5) by incubation overnight at 4°C. The coated plates were washed with 25 mM potassium phosphate buffer pH 7.4, 0.15 M NaCl and free sites were blocked by incubation in 50 mM Tris, 150 mM NaCl, 2% Tween 20 for 1 h at 37°C. Inhibitors (10^−6^ M) were incubated in these wells with TGase (1.25×10^−7^ M) or FXIIIa (10^−6^ M) in 50 mM Tris-HCl buffer, pH 7.5, 2 mM CaCl_2_, 0.1 mM DTT for 2 h at 37°C to form cross-linked complexes with fibronectin or elastin. The plates were again extensively washed with the same buffer as above and human elastase (10^−9^ M), proteinase 3 (2×10^−9^ M), or cathepsin G (2×10^−9^ M) in appropriate buffers was added and incubated for 15 min at 37°C to form protease-inhibitor complexes. Residual protease activity was then measured with the appropriate fluorogenic substrate (10 µM) and a SPECTRAmax Gemini microplate reader (Molecular Devices France, St Grégoire, France). Data are represented as means of residual enzymatic activity ±SEM and were analyzed using the analysis of the variance (ANOVA) followed by post hoc Student-t test (***p≤0.001, **p≤0.005 and *p≤0.05).

### Mass spectrometry analysis

#### MALDI-TOF mass spectrometry on whole proteins

All mass spectra were generated on a M@LDI LR (Waters, Manchester, UK) MALDI-TOF mass spectrometer, operating in positive linear mode. Aliquots (0.5 µL) of sample and matrix (1∶2, v/v) (20 mg/ml sinapinic acid) in 50% acetonitrile, 0.1% TFA were loaded on the target using the dried droplet method. External mass calibration was performed with a mixture containing Glu-fibrinopeptide B, ACTH (18–39 clip), insulin, ubiquitin (all at 1 pmol/µL), cytochrome C (2 pmol/µL), myoglobin (4 pmol/µL) and trypsinogen (8 pmol/µL). Mass spectra were recorded in the mass range 1000–30000 *m/z*, acquiring 10 shots per spectrum at a laser firing rate of 10 Hz. Data were processed using MassLynx™ 4.0 software. The background was subtracted from the spectrum from each sample well using a polynomial order of 10% below the curve and smoothed with the minimum peak width at half height set to 15 channels. Two smoothes were performed using the Savitzky Golay algorithm. Internal mass calibration was performed with Lock Mass option at m/z 9930.78 for trappin-2, 6000.26 for elafin and 11711.05 for SLPI. All spectra were processed using the same parameters.

#### MALDI-TOF peptide mass fingerprint

Each sample were reduced with 5 mM dithiothreitol, alkylated with 12.5 mM iodoacetamide and incubated overnight at 37°C with 0.1 µg bovine trypsin (Roche, Paris). The tryptic digests were acidified with 1 µL 5% formic acid in water and sonicated for 10 min. The matrix used was 5 mg/mL α-cyano-4-hydroxycinnamic acid in 50% ethanol/50% acetonitrile/TFA 0.1%/10 mM crown ether18-C6. The sample and the matrix (1∶1, v/v) were loaded onto the target using the dried droplet method. MALDI-TOF spectra of the peptides were obtained with M@LDI L/R P/N mass spectrometer (Waters, Manchester UK) in the 500–4500 m/z mass range. The analyses were performed in positive ion reflectror mode acquiring 10 shots per spectrum at a laser firing rate of 5 Hz, with an accelerating voltage of 15000 V. The resulting spectra were calibrated externally using the [M+H]^+^ ions from a bovine serum albumin digest. Raw data files were converted to mzXML with ProteinLynx Global server 2.2 software (Waters, Manchester, UK). MS data were matched automatically to a database containing the trappin-2, elafin and SLPI protein sequences using the Peptide Mass Fingerprint search option of the MASCOT server software (Matrix Science, UK). Enzyme specificity was set to trypsin with 5 missed cleavages using carbamidomethylcysteine (+57 Da), methionine oxidation (+16 Da), deamidated N/Q (+1) and Dsc (+318 Da) or PQQGGIV (+680 Da) as variable modifications. The tolerance of the ions was set to 150 ppm.

#### NanoLC-MS/MS

The Ettan MDLC controlled by UNICORN™ software (GE Healthcare, Germany) was used to desalt and separate tryptic peptides prior to online MS and MS/MS analyses. Aliquots (4 µL) of digested sample were mixed with 10 µL 1% formic acid and 10 µL of these mixes were injected using the µL-pickup mode. Each sample was automatically desalted and concentrated using a Zorbax 300-SB C_18_ trap column, 300 µm i.d×5 mm (Agilent Technologies, Germany). Peptide were separated on a Zorbax 300-SB C_18_ column, 75 µm i.d×150 mm (Agilent Technologies, Germany). Buffer A was 0.1% formic acid in water and buffer B was 0.1% formic acid in 84% aqueous acetonitrile. Peptides were eluted with a 15–55% gradient of buffer B at a flow rate of 350 nL/min for 60 min. The peptides were analysed online with an LTQ Linear Ion Trap Mass Spectrometer (Thermo Electron, US) using a Thermo Electron Dynamic Nanospray Probe interface. Ionisation was performed (1.8–2.1 kV) with liquid junction and uncoated fused-silica nanoESI 25 µm i.d emitters (New objective, Woburn, USA). The ion transfer capillary was set to 200°C. Each scan cycle consisted of one full scan mass spectrum (m/z 500–2000) collected in enhanced mode followed by three MS/MS events in centroid mode (Qz = 0.25, Activation Time = 40 ms). The isolation width for CID spectra (MS2) was 2 m/z units and the normalized collision energy was 40%. Dynamic exclusion was activated for 30 s with a repeat count of 1. Raw data files were converted to mzXML with Bioworks 3.3.1™ software (Thermo Fischer Scientific, San Jose, CA). The peptide and fragment masses obtained were matched automatically with a database containing the trappin-2, elafin and SLPI protein sequences and using the MASCOT Daemon v2.2.2 (Matrix Science, UK) search option of the MASCOT server software. Enzyme specificity was set to semitrypsin with 5 missed cleavages using carbamidomethylcysteine (+57 Da), methionine oxidation (+16 Da), deamidated N/Q (+1) and Dsc (+318) or PQQGGIV (+680) as variable modifications. The tolerance of the ions was set to 1.4 Da for parent and 1.0 Da for fragment ion matches. Hits with a P value<0.05 were manually verified and peptides were considered positively identified with 5 consecutive fragment ions.
